# Decoding the infant mind: Multivariate pattern analysis (MVPA) using fNIRS

**DOI:** 10.1371/journal.pone.0172500

**Published:** 2017-04-20

**Authors:** Lauren L. Emberson, Benjamin D. Zinszer, Rajeev D. S. Raizada, Richard N. Aslin

**Affiliations:** 1 Psychology Department, Princeton University, Princeton, NJ, United States of America; 2 Brain and Cognitive Sciences Department, University of Rochester, Rochester, NY, United States of America; 3 Rochester Center for Brain Imaging, University of Rochester, Rochester, NY, United States of America; Centre de neuroscience cognitive, FRANCE

## Abstract

The MRI environment restricts the types of populations and tasks that can be studied by cognitive neuroscientists (e.g., young infants, face-to-face communication). FNIRS is a neuroimaging modality that records the same physiological signal as fMRI but without the constraints of MRI, and with better spatial localization than EEG. However, research in the fNIRS community largely lacks the analytic sophistication of analogous fMRI work, restricting the application of this imaging technology. The current paper presents a method of multivariate pattern analysis for fNIRS that allows the authors to decode the infant mind (a key fNIRS population). Specifically, multivariate pattern analysis (MVPA) employs a correlation-based decoding method where a group model is constructed for all infants except one; both average patterns (i.e., infant-level) and single trial patterns (i.e., trial-level) of activation are decoded. Between subjects decoding is a particularly difficult task, because each infant has their own somewhat idiosyncratic patterns of neural activation. The fact that our method succeeds at across-subject decoding demonstrates the presence of group-level multi-channel regularities across infants. The code for implementing these analyses has been made readily available online to facilitate the quick adoption of this method to advance the methodological tools available to the fNIRS researcher.

## Introduction

The goal of cognitive neuroscience is to use the relationship between activity in the brain and cognitive operations to understand how the mind works. In the last two decades, the use of fMRI has vastly expanded our window on the neural correlates of human cognition. Initially, fMRI analyses predominantly facilitated brain mapping: Experiments could tell us where in the brain clusters of voxels show differential BOLD signals to two or more stimulus conditions. With the addition of multivariate analysis techniques (e.g., multivoxel pattern analysis, MVPA), more sophisticated questions can be asked, such as whether the *pattern* of BOLD can discriminate between two or more stimulus conditions. Multivariate analyses are an important advance as they have shifted the focus of cognitive neuroscience from mean activation differences to the information contained within patterns of brain activity (see [1] for an example).

MVPA studies have provided compelling evidence that the adult brain contains distributed patterns of neural activity [2–4]. Studies have demonstrated the power of MVPA to decode natural visual images [5], noun identity [6], and speaker identity [7]. Indeed, the use of multivariate methods has become widespread, with thousands of papers employing this method with fMRI data. Analogous multivariate methods having also been developed for EEG (e.g., [8]), MEG (e.g., [9]), and intracranial recordings (e.g., [10])

However, despite fMRI’s power to address foundational questions in cognitive neuroscience, it is not universally applicable to all kinds of subject populations. For example, early developmental populations (e.g., infants) have not yet been successfully scanned while awake. Many clinical populations such as those with acute anxiety disorders, movement disorders, or cochlear implants also cannot participate in MRI experiments. Furthermore, the scanner environment greatly restricts the types of cognitive tasks and abilities that can be investigated. Cognitive abilities, such as face-to-face communication or motor movements, cannot be assessed while laying supine and motionless inside the tightly enclosed and noisy bore of the scanner. Thus, the MRI environment drastically restricts the kinds of tasks and populations that can be studied.

**Fig 1:**
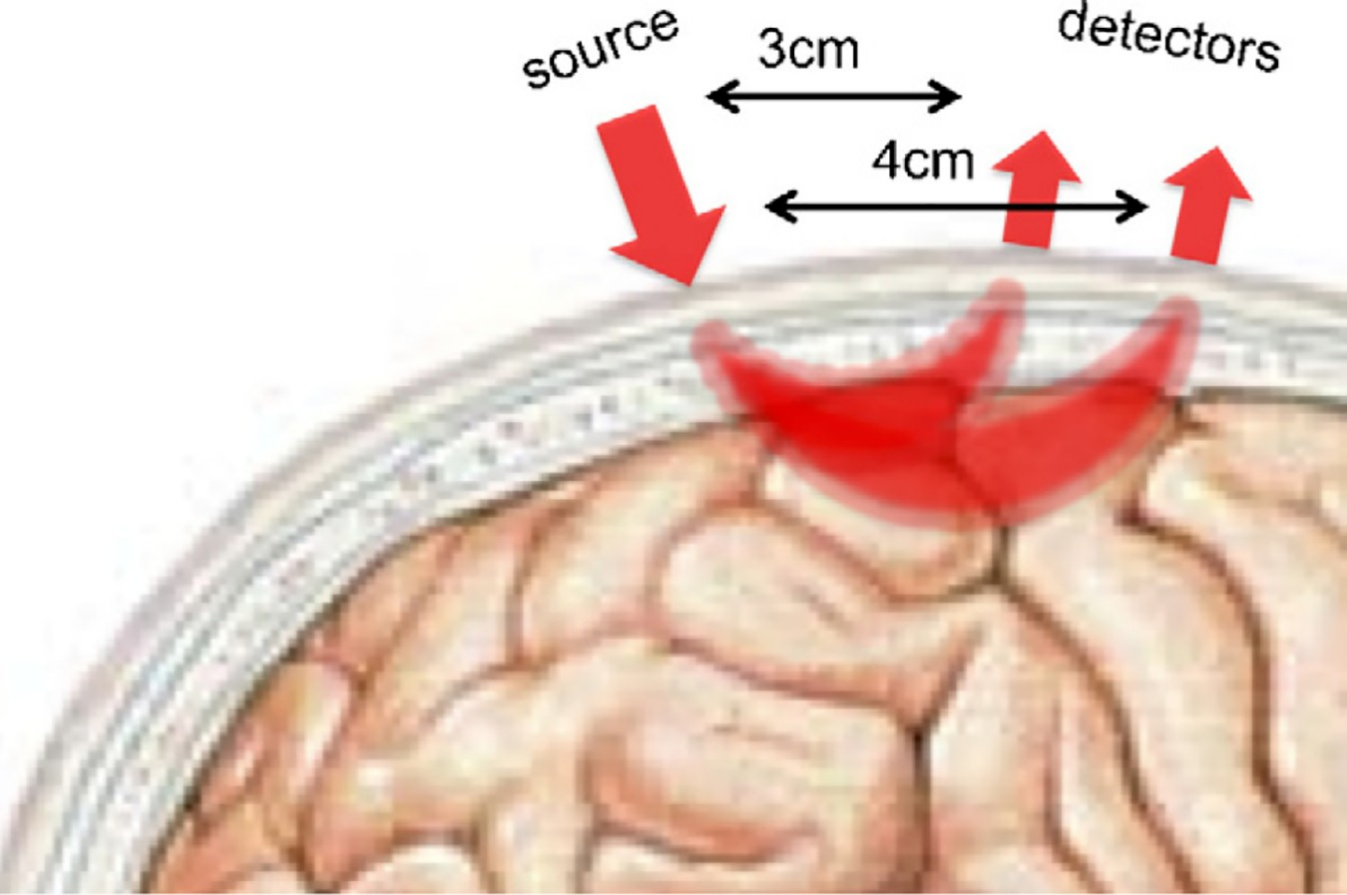
Functional near-infrared spectroscopy (fNIRS) records cortical hemodynamic responses in populations that cannot comfortably be inside the MR scanner such as young infants. Pairs of detectors and emitters form an fNIRS channel (from Gervain et al., 2011 with permission) which covers a localizable region of the cortex.

Functional near-infrared spectroscopy (fNIRS) is a complementary neuroimaging modality that has gained popularity in recent years for its ability to deal with many of these constraints that limit the use of fMRI. The fNIRS device records the same physiological substrate as fMRI (i.e., changes of blood oxygenation in the cortex arising from neurovascular coupling) using scattered infrared light instead of magnetic fields and radio waves. Specifically, fNIRS requires participants to wear a cap embedded with detectors and emitters of near-infrared light, similar to a pulse oximeter. A detector-emitter pair forms a *NIRS channel* within which cortical hemodynamic responses can be recorded (Fig 1). Crucially, this method for recording the hemodynamic response is free from requirements that make the MRI environment so restrictive. No magnetic fields are needed, and the device is not sensitive to local electrical interference. Thus, it is safe for participants with metal or magnetically sensitive implants, and the machine is highly portable to many types of environments. The fNIRS device is silent, the cap is relatively comfortable to wear, and the measurements are more robust to movement than MRI. A different imaging modality, EEG, also has the advantages of being quiet and using sensors that can be attached directly to the head. However, fNIRS has significantly better ability to spatially localize neural signals than does EEG, which has poor localization due to the conduction of the electrophysiological signals throughout the head. In other words, although the coverage of a single fNIRS channel is large, one can be highly confident that the signal is specific to that region.

While there are clear limitations to fNIRS (e.g., the absence of an anatomical image, lower spatial resolution, and the ability to record only from the surface of the cortex), it provides an important tool to expand cognitive neuroscience into areas of inquiry previously beyond our methodological reach (see recent reviews from a developmental perspective: [11,12]).

A major hurdle to the widespread acceptance of fNIRS, as an alternative to fMRI, fNIRS data analysis techniques have historically lagged behind fMRI in sophistication. Part of this analytic immaturity arises from inherent methodological limitation of fNIRS. For example, NIRS systems do not collect the anatomical images that enable fMRI signals to be spatially localized (see [13] for recent advanced in this area). However, other aspects of fNIRS data analysis have fallen behind for reasons not inherent to the method. For example, only recently have fNIRS researchers employed corrections for multiple comparisons (see [14] for a review of the history of statistical techniques with fNIRS) or defined prior neuroanatomical regions of interest validated by structural MRI templates [15,16]. While fNIRS methodology has greatly progressed, this neuroimaging modality will only reach its potential as a complementary neuroimaging technique if it achieves a high level of analytical sophistication.

In this paper, we propose an important step forward for fNIRS data analysis by presenting a simple, easily implemented method for conducting multivariate pattern analyses with fNIRS data gathered from infants. Several comprehensive tutorials have explored the application of machine-learning and multivariate methods to fMRI data [17,18], but after 15 years of fMRI-based decoding, fNIRS still lacks a comparable literature. We aim to begin closing this gap. Our application of MVPA to fNIRS is computationally fast and immediately applicable to a variety of preprocessing routines already used in the fNIRS field. Moreover, we’ve made the code to implement these analyses freely available, along with the data`sets used in the current paper (http://teammcpa.github.io/EmbersonZinszerMCPA/ and http://dataspace.princeton.edu/jspui/handle/88435/dsp01xs55mf543). The code can be used as a modular function with a variety of other data sets or can be readily adapted to future applications.

The application of MVPA to fNIRS data has the potential to extract substantially greater detail about the neural correlates of cognition than can be revealed using univariate statistics. On one hand, multivariate methods can be more sensitive: Indeed, prominent results in the fMRI literature have found that hemodynamic responses encode significant information about participants’ cognitive states without producing a robust univariate contrast (e.g., [1,19]). In the present study, we demonstrate this same sort of result for the first time using fNIRS in infants: as is described in more detail below, we show that when the classifier’s task is to distinguish between two conditions which are both audio-visual but which differ in the specific nature of the audio-visual stimuli (faces-and-music, versus fireworks-and-speech) our multichannel decoding approach is able to succeed whereas purely univariate analysis fails.

However, considering multiple channels at once yields more important benefits than simply being more sensitive than univariate analyses. A more interesting distinction is the fact that although univariate activation intensity can only go up or down, multivariate patterns have similarity relations to each other, and therefore induce a structured similarity space (see Representational Similarity Analysis or RSA, [20,21]) Similarity measures can, for example, quantify how much a new observation matches previous observations and thus be used to classify the new observation. These sorts of questions of representational structure simply do not arise in a purely univariate framework and thus provide an opportunity for greater detail rather than a more sensitive contrast between two conditions.

Despite the success of multivariate methods for neural decoding in other imaging modalities, there are significant challenges to applying these methods to fNIRS. First, while fNIRS records local and specific hemodynamic changes in the cortex (like fMRI), the number of recording sites is typically very few (e.g., 10 to 25 channels). Second, the spatial resolution is greatly decreased relative to an fMRI voxel: A typical channel measures an approximately 2 cm^2^ region on the surface of the cortex. The spatial distribution of these recording sites across the scalp is also usually sparse, with a 2 cm separation between adjacent channels, and signals are not sampled any deeper than 2 cm into the cortical tissue. Thus, on the one hand, the number of fNIRS recording sites is similar to EEG (on the order of dozens). On the other hand, fNIRS records a spatially localized signal, unlike EEG. Although it may be difficult to conceive of successful MVPA decoding with only 10–25 “super” voxels sparsely distributed over the cortex, these are the most typical conditions under which multivariate methods are likely to be applied to fNIRS data.

The amount of data gathered within- and across-participants also significantly differs between fNIRS and other imaging modalities. Many populations that can be readily studied with fNIRS but not fMRI (e.g., infants) cannot participate for the large number of trials typically seen in MVPA designs. These fNIRS datasets typically have a greater number of participants than the average number of trials per participant. Consequently, while many fMRI MVPA studies focus on within-subject decoding techniques and avoid the problems associated with across-subject generalization, it is unclear whether decoding will be successful with the small number of trials and the high-degree of between-subject variability typical of fNIRS datasets. Thus, while multivariate methods have achieved broad success within the fMRI community (and increasingly in high-density EEG and MEG), it is unclear whether this approach will be successful given the constraints of the typical infant fNIRS dataset.

While there have been a few successful attempts applying multivariate methods to fNIRS data from adults or children, no previous study has attempted the far more challenging task of decoding fNIRS data from infants. This is likely because infant datasets always contain fewer stimulus-presentation trials and more noise than the data that can be obtained from older and hence more cooperative participants. The present study employs some similar approaches used in this prior body of work, but they also differ in crucial ways. Previous studies have decoded emotional state [22], subjective preference [23,24], item price [25], vigilance [26], and ADHD and autism diagnoses [27] from fNIRS recordings. While the results are promising (decoding accuracy between 60–70%), these initial attempts fall short of providing a methodological foundation from which the fNIRS community can build further multivariate analyses. First, these studies have all employed Support Vector Machine (SVM) algorithms for classification. SVMs are a machine learning technique that seek a set of weights for the hemodynamic signal from each voxel or channel that best predicts classification accuracy with as large a margin as possible between the classes to be distinguished [2].

Several alternate classification methods have also been successfully applied to decoding fMRI data and may prove useful for multivariate fNIRS analyses as well. Similarity-based decoding is one such example. This approach uses representational similarity structures (RSA, described above) to classify new observations based on their similarity to other known observations in the similarity structure [21]. A very simple version of an RSA approach is simply using Pearson correlation, and we explore the application of this method to infant fNIRS data. Specifically, we will explore whether Pearson correlation can be used to show that infants’ neural responses to similar stimuli (within Condition) are relatively homogenous compared to neural responses to dissimilar stimuli (across Condition). If a correlation-based decoding was successful, RSA-based decoding would likely succeed in future infant fNIRS studies with multiple stimulus classes.

Second, these previous studies typically assessed adults rather than the infant and clinical populations that are uniquely suited for fNIRS imaging. The current limitation of multivariate fNIRS methods to typically functioning adults has thus not inspired broader application. Many populations typically studied in fNIRS cannot contribute a large number of trials in an experiment and are more difficult to test. Thus, these studies (with exception of [27]) have larger pools of data from each individual participant available for the classification (typically 50+ samples per participant) but these large within-subject datasets would be extremely difficult to achieve in infants or many clinical populations. Ichikawa et al [27] overcame this limitation to decode clinical diagnoses (ADHD/autism) in children with a classifier that required a large amount of computational power. The training necessary for this technique required the calculation of 2^24^ or 17 million subsets of the data. Given the great deal of neural variability across individuals, especially in clinical populations and early in development, performing decoding across individuals is quite difficult and has typically been more challenging for MVPA studies using fMRI. However, as we will demonstrate, across-infant multivariate inferences can produce highly accurate results while overcoming one of the most common practical limitations of infant research (limited numbers of trials per infant).

The current study extends previous work in three major respects:

1. We employ a correlation-based decoding method that is computationally simple, fast, and easily interpretable. The code for implementing all the analyses and a sample dataset are included to encourage immediate application and broad adoption in the fNIRS community.
2. The present study is the only reported multivariate analysis with infants. A large number of researchers employing fNIRS are doing so to probe the neural correlates of early development, but they have exclusively used univariate methods [12], It is currently unknown whether decoding the infant brain is possible using MVPA, especially given the small number of trials contributed by infants in a typical experiment.
3. The current MVPA analysis performs all classifications *across* infants. Data from an infant who does not contribute to the group model are classified by aggregating data across other infants in the dataset. The between-subjects approach takes advantage of the larger number of available participants in an experiment than trials completed by each participant.

Our overall goal is to determine whether MVPA can decode the infant brain. Specifically, we examine whether patterns of activation across multiple NIRS channels can accurately predict the patterns of activation in an infant who did not participate in the creation of a *group model*. We examine the decoding accuracy for each *test infant*’s activation patterns as that infant’s data are iteratively removed from the group model. We refer to the resulting classification of each test infant’s condition averages as *infant-level* decoding. We also examine the much more difficult task of classifying each of the test infant’s individual trials (*trial-level* decoding) based only on the group model to which the left out test infant did not contribute.

In sum, we ask whether a group model of neural responses created from several infants sufficiently generalizes to classify observations from a new infant (the test infant). Our measure of success is whether decoding produces accurate predictions (i.e., significantly above chance) when these MVPA methods are iterated across the entire sample of infants. To determine whether this method is robust and generalizable to new experiments, we will also apply it across two separate groups of infants in different experiments presenting different levels of decoding difficulty.

## Materials and methods

Our application of MVPA to fNIRS uses a group model, derived from multichannel patterns of activation, to decode unlabeled patterns of activation that did not contribute to the group model. We begin by describing how ***multichannel patterns*** are calculated, then how the ***group model*** is created, and finally how this group model is used to decode ***unlabeled*** multichannel patterns. All analyses were conducted in MATLAB (version R2013a, 8.1.0.604) with custom analysis scripts.

**Fig 2:**
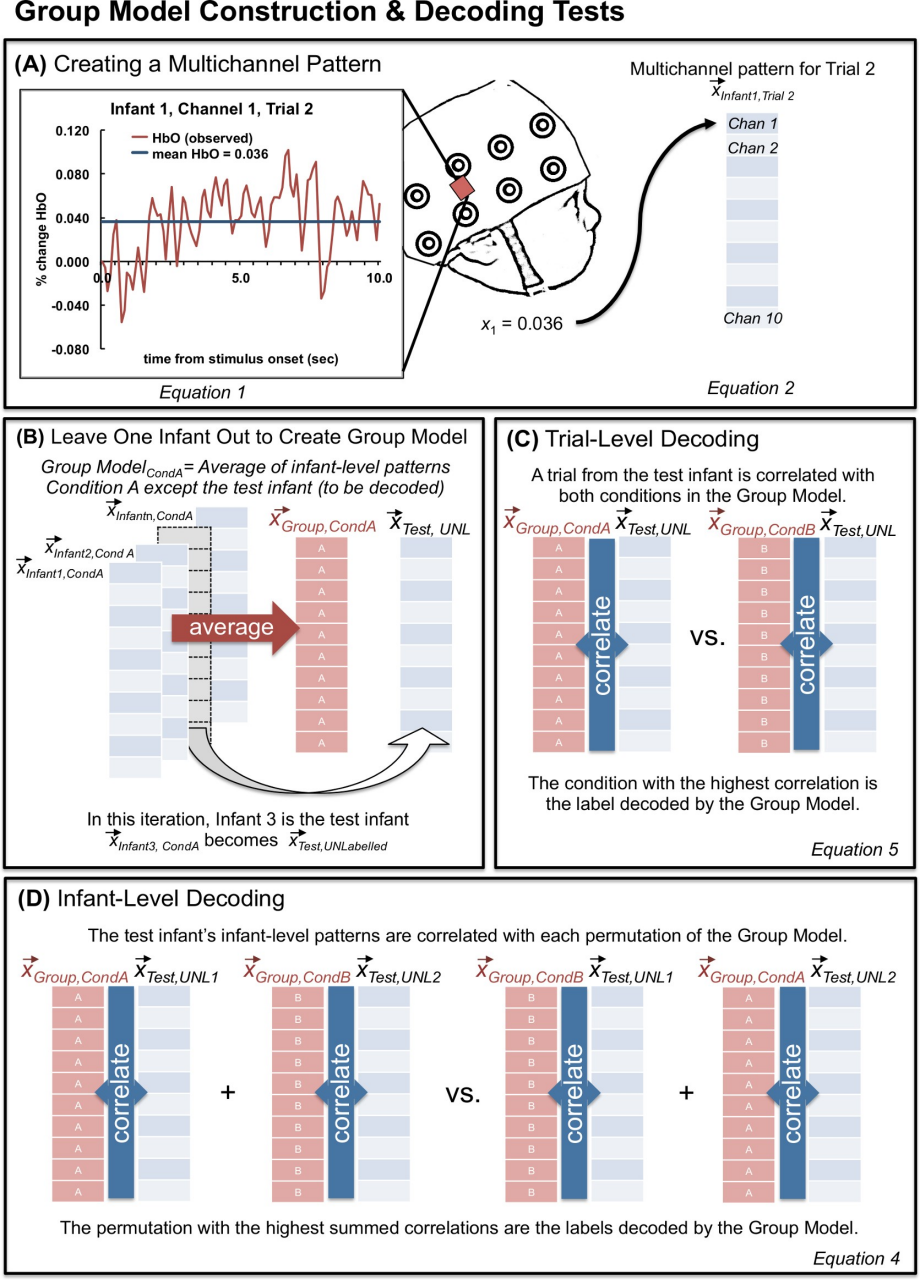
Illustration of the multivariate methods applied to fNIRS in this paper.

First, multichannel patterns are constructed by including information for all channels, for each trial, for every infant. MVPA treats each channel as an independent observation. To derive a multichannel pattern, oxygenated hemoglobin (HbO) is averaged over an *a priori* determined time-window (i.e., up to *t* measures after stimulus onset) for each channel (*chan*). This measure is re-baseline corrected to the first measurement. This is described in Eq 1 and Fig 2A.

$$x_{chan}=\frac{1}{t}\sum_{i=1}^{t}(HbO_{chan,i}-HbO_{chan,1})$$ (1)

It is standard in the developmental fNIRS literature to focus on the oxygenated response (see recent review: [11]), however, there would be no change in the method to conduct this analysis with the deoxygenated response. Extensions of this method would be required to include both the oxygenated and deoxygenated responses simultaneously. The average oxygenated response (*x*_*chan*_) is determined for each channel for the same time-window and then combined with the corresponding averages from the other channels into a single vector to create a *multichannel pattern* (*x*) for a single time-window (e.g., single trial), for a single infant (see Fig 2A). These multichannel patterns are vectors of dimension *n* where *n* equals the number of channels.

$$\overset{⃑}{x}=[x_{1},x_{2},\ldots x_{n}]$$ (2)

Multichannel patterns can then be averaged across all the trials (*r*) of a given stimulus condition within infants to give rise to an ***infant-level multichannel pattern*** for that stimulus condition (i.e., in an experiment with two conditions, two such vectors are calculated, one for each condition).

$${\overset{⃑}{x}}_{Infant,Cond}=\frac{1}{r}\sum_{j=1}^{r}{\overset{⃑}{x}}_{Infant\;n,Cond\;A,Trial\;j}$$ (3)

To construct the ***group model***, infant-level multichannel patterns, for each stimulus condition, are averaged to produce a group-level multichannel pattern (*x*_*Group*,*Cond*_). However, it is important to note that not all infants are included in this average: we employ a leave-one-out method so a single ***test infant*** is removed from the average and the group model is created from *N*-1 infants for the current experiment. See Fig 2B. This leave-one-out process is iterated N times so that each infant has been left out and will serve as the test infant, and thus, the group average has been recomputed N times from the remaining (non-test) infants.

The multichannel patterns are compared to one another using Pearson correlation. Because the multichannel pattern vectors have the same number of dimensions (the number of channels) at all levels of analysis, we can compare a group-level model to (1) the infant-level multichannel pattern for the test infant or (2) the multichannel patterns for single trials from the test infant. We will refer to the former as ***infant-level decoding*** and the latter as ***trial-level decoding***. While these two types of decoding are derived from the same basic principles, they differ in that the infant-level decoding has multiple infant-level multichannel patterns to decode simultaneously (the number is equivalent to the number of stimulus conditions, but in this paper, we compare across two conditions exclusively), whereas trial-level decoding, by definition, has only a single multichannel pattern to decode. These differences in methodology between the two types of decoding result in subtle differences in how the group model is used to decode these multichannel patterns of activation. We now describe these differences in detail.

Infant-level decoding uses the group model to predict the infant-level multichannel patterns of the test infant for each stimulus condition. Specifically, the test infant’s multichannel patterns are decoded by determining which permutation of condition labels yields the greatest overall correlation to the group-level model. For two conditions, A and B, two Pearson correlations are computed as follows:

$$\tanh^{-1}\left(corr(x_{Group,CondA},x_{Test,Unl1})\right)+\tanh^{-1}\left(corr\left(x_{Group,CondB},x_{Test,Unl2}\right)\right)$$ (4.1)

&

$$\tanh^{-1}\left(corr(x_{Group,CondB},x_{Test,Unl1})\right)+\tanh^{-1}\left(corr(x_{Group,CondA},x_{Test,Unl2})\right)$$ (4.2)

The sums of the Fisher r-to-z (hyperbolic arctangent) transformed Pearson correlations are compared. Decoding is considered successful if the sum of the correlations with the correct labels is greater than the sum of the correlations with the incorrect labels. In other words, if Unlabeled1 is the test infant’s average response to ConditionA and Unlabeled2 is the test infant’s average response to Condition B, one could consider that whichever permutation yields the greatest sum of correlations (A-1, B-2 vs. A-2, B-1) provides the best estimate of labels for the new, unlabeled responses. If the new labels are correct, then decoding of this test infant with the group model is considered accurate, and if not, then decoding is considered inaccurate (a 1 or a 0 is assigned for this test infant, respectively).

For trial-level decoding, the test infant’s multichannel patterns for each unlabeled trial are correlated with each condition from the group model. If the correct label yields the greater correlation then we consider decoding to be successful. For example, a correctly decoded ConditionA trial would be represented by the following equation:

$$\tanh^{-1}\left(corr(x_{Group,CondA},x_{Test,Unl})\right)>\tanh^{-1}\left(corr(x_{Group,CondB},x_{Test,Unl})\right)$$ (5)

After binary decoding accuracy (1/0) is recorded for each trial for a given test infant, average accuracy for each condition is derived by averaging decoding accuracy for all trials of a given condition.

Once decoding (infant-level or trial-level) is complete for a given test infant, this procedure is iterated throughout the population of infants until each infant has been a test infant and their patterns of activation have been decoded. Specifically, after decoding is complete the test infant is reintegrated and the multichannel patterns from another infant are removed (i.e., the new test infant) before a new group level model is created. To conduct decoding for *N* infants in a given experiment, *N* group models are created with a unique model for each infant.

These methods can be applied to three or more stimulus conditions, but the present study will focus on the case of only two conditions. Extension to a greater number of conditions is further addressed in the Discussion.

### Application of MVPA to infant fNIRS data

We applied our MVPA method to two previously collected infant fNIRS datasets. Both datasets were obtained from a Hitachi ETG-4000 and a custom cap and optical fibers built especially for fNIRS data collection with infants. FNIRS data were sampled at 10Hz. Twenty-four channels were used in the NIRS cap, with 12 over the back of the head to record bilaterally from the occipital lobe, and 12 over the left side of the head to record from the left temporal lobe. The channels were organized in two 3x3 arrays, and the cap was placed so that, for the lateral array, the central optode on the most ventral row was centered over the infants’ left ear and, for the rear array, the central optode on the most ventral row was centered between the infant’s ears and over the inion. This cap position was chosen based on which NIRS channels were most likely to record from temporal and occipital cortex in infants. Due to curvature of the infant head, a number of channels did not provide consistently good optical contact across infants (the most dorsal channels for each pad rarely if ever made physical contact with the infant’s scalp and thus were *a priori* excluded for the entire population). We did not consider the recordings from these channels in subsequent analyses and only considered a subset of the channels (7 for the lateral pad over the ear and 3 for the pad at the rear array).

Written consent was obtained from a legal guardian for each infant before the experiment. This consent procedure and the experimental methods were approved by the Institutional Review Board of the University of Rochester. During the experiment, the infant sat on a caretaker's lap in a darkened room and surrounded by a black curtain to reduce visual distraction and to separate the participant from the experimenter. Caretakers were instructed to refrain from influencing their infant, only providing comfort if needed. Infants watched the video until they consistently stopped looking, became fussy, or, in the case of dataset #2, all experimental blocks were watched.

Raw data were exported to MATLAB (version 2006a for PC) for subsequent analyses with HomER 1 (Hemodynamic Evoked Response NIRS data analysis GUI, version 4.0.0) for a standard preprocessing of the NIRS data. First, the “raw intensity data is normalized to provide a relative (percent) change by dividing the mean of the data” (HomER 1.0 manual). Then the data is low-pass filtered (cutoff 3 Hz) to remove noise such as heart-beat. Second, changes in optical density are calculated for each wavelength, and a PCA analysis was employed to remove motion artifacts. Finally, the modified Beer-Lambert law is used to determine the changes (delta) concentration of oxygenated and deoxygenated hemoglobin for each channel (the DOT.data.dConc output variable was used for subsequent analyses, see the HomER Users Guide for full details [28]).

### Dataset 1: Differentiating auditory and visual processing

**Fig 3:**
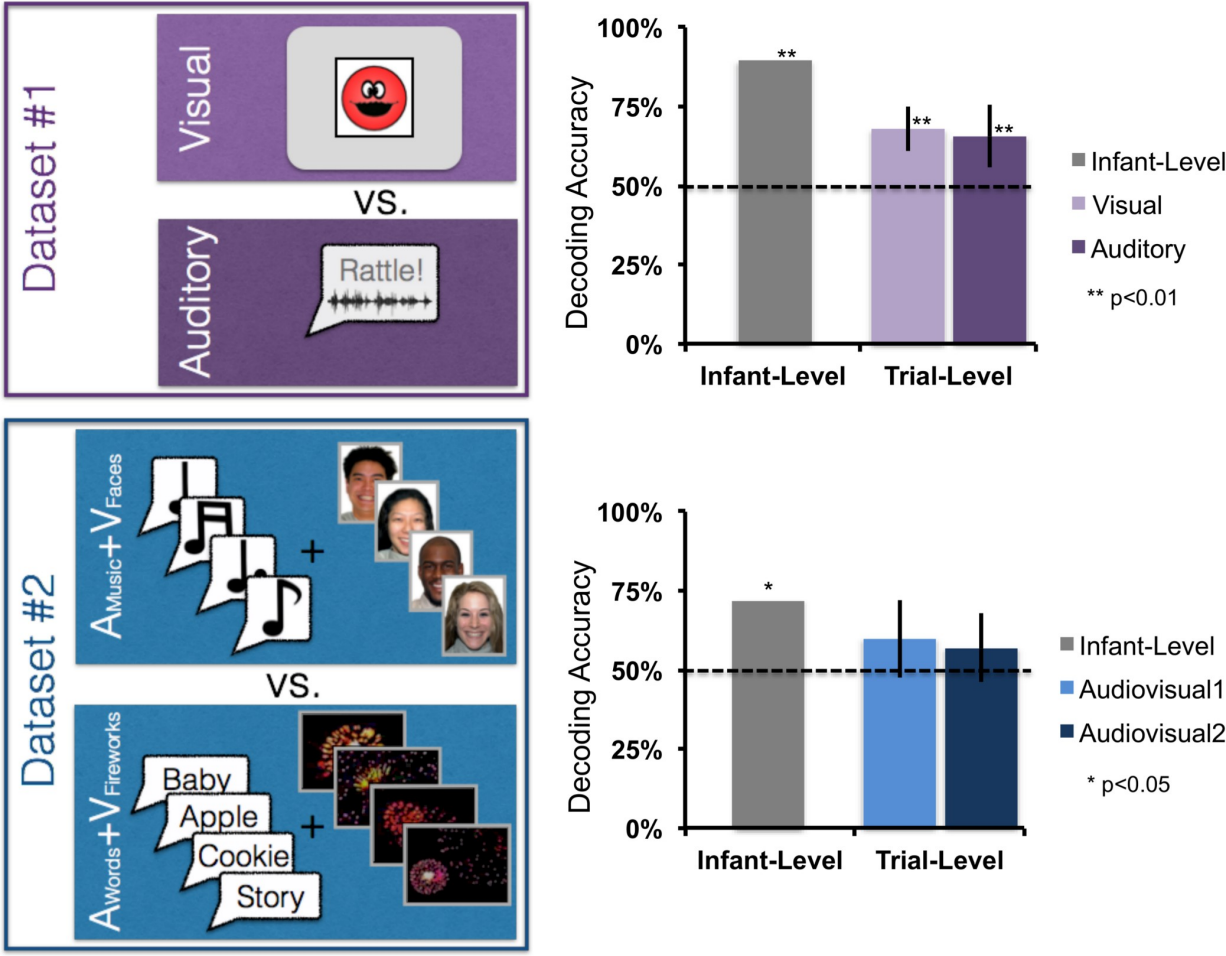
Depiction of the two datasets and the decoding results (infant-level and trial-level) for each. Error bars depict the bootstrapped confidence intervals of the mean across infants.

This dataset came from an experiment in which infants either viewed a visual stimulus (a red smiley face appearing for 1 sec in a white box on the screen) or heard an auditory stimulus (a toy sound, either a rattle or a honk sound that played for 1 sec) in separate events in an event-related design. Including static presentation of the empty white square on either side of these unimodal stimulus events, these trials lasted 3–3.5 seconds (see 15 for detailed information about stimulus presentation) and were separated by a jittered baseline lasting between 4–9 seconds (mean 6.5 seconds). See Fig 3 (left panel) for a schematic of this task (i.e., which information we must decode from the neural signal).

Twenty-five (25) infants were recruited for this study (mean age = 5.7, *SD* = 0.61 months, 10 female, 2/25 infants were identified as Hispanic and 23 infants were identified by their parents as Caucasian and 2 were identified as mixed race, Caucasian + Asian, Caucasian + Native American + Black). Of these infants, 19 were included in the final data analyses, with 3 infants excluded due to poor optical contact (e.g., due to a large amount of dark hair; these individual subject exclusions were made based on experimental notes or from observation of the recordings. The decision to include or exclude each subject was made once and before the data were analyzed in order to reduce the possibility for experimental bias) and 3 for failing to watch the video to criterion. Infants were recruited through the database of interested participants from the Rochester Baby Lab and were born no more than 3 weeks before their due date, had no major health problems or surgeries, no history of ear infections, nor known hearing or vision difficulties. Caregivers were compensated $10 for their visit and a token gift (e.g., a Baby Lab t-shirt, bib or tote bag).

Separate univariate analyses for Dataset 1 were previously reported in [29]. For more details, please refer to that manuscript. On average, infants watched for 6.9 trials of each of the two unimodal conditions (*SD* = 1.95). For the current MVPA analyses, the data were analyzed with a time-window of 0–10 seconds. This is a different time-window than that employed in Emberson et al. (2015), which employed a 4–11.5 second time-window. This difference reflects the fact that Emberson et al. [15] used univariate analyses to capture the peak of the hemodynamic response function, whereas the current analysis it was important to maximize the variability of the response curve for optimal decoding. Thus, a time-window was chosen which included the rise of the hemodynamic response in the first four seconds. Parameters for selecting the optimal time window for a particular experiment are not yet clear, so while this visual inspection approach proves sufficient in the present study, the same time window may not generalize to all event-related designs. In the supplementary materials (S1 File), we review the consequences of this time window selection relative to other possible windows. Future research will be necessary to evaluate the impact of time window parameters for decoding other datasets and the best methods for selection.

### Dataset 2: Differentiating combinations of audiovisual processing

In this dataset, infants either viewed a block of 8 faces while listening to music (**V**_**Faces**_**+A**_**Music**_) or they listened to a block of 8 words while looking at dimmed fireworks (**V**_**Fireworks**_**+A**_**Words**_). Thus, in this dataset, successful decoding of the pattern of neural responses required distinguishing between types of audiovisual (AV) processing and not simply whether the infant is hearing something or seeing something as in Dataset #1. The audio stimuli were 8 common words familiar to infants and commonly attested in samples of infant-directed speech (apple, baby, bottle, blanket, cookie, diaper, doggie, story). The visual face stimuli were 8 smiling Caucasian female faces from the NimStim database [30]. All stimuli had a stimulus onset interval of 1 second. The inter-stimulus interval (ISI) for visual stimuli was always .25 seconds. The ISI for audio stimuli ranged from .2-.3 seconds because of slight differences in word duration. The 8 stimuli in each modality were presented in shuffled order for each block. As with Dataset #1, these stimuli were followed by a jittered 4–9 second baseline (mean = 6.5 sec). See Fig 3 for a schematic of this dataset. Unrelated univariate analyses of this dataset are presented in [31] with more details on the experiment available in that manuscript.

Twenty-six infants were recruited based on the same criteria and using the same methods as Dataset #1. Of these, 18 infants were included in the final analysis: Infants were excluded for poor optical contact (6, see above), not watching to criterion (1), or refusing to wear the NIRS cap (1). The remaining sample of infants had a mean age of 5.8 months (*SD* = 0.6) and consisted of 9 females and 9 males. Of the included infants, 88.9 percent heard only English at home. Two other participants heard another language from their family 60 or 90 percent of the time. Participants were identified as Caucasian (16), black (1), and Hispanic (2). Infants watched these two multimodal blocks an average of 4.9 times each (*SD* = 1.17). As with Dataset #1, the time-window investigated for MVPA is slightly different than for the corresponding univariate analyses (6–14.5 seconds, 30). In this block design, our analysis focused on the cumulative response to the eight stimuli during the exposure period. Thus the current analyses employ a time window of 6–12.5 seconds during which the fNIRS response was likely to plateau. Individual trials may vary in the first 6 seconds after stimulus onset while the fNIRS response is still rising, but the plateau is more likely to stabilize throughout the course of the block. Similar to Dataset #1, this time window also curtails the end the hemodynamic response period. As we are decoding over averaged windows of oxygenated hemoglobin, in general, there is not yet a clear and principled way to select time-windows for the current analyses. Future work could identify optimal timing for event designs (as in Dataset 1) versus block designs (as in Dataset 2) or model beta values as in adult fMRI analyses. The application of multivariate methods to infant fNIRS data is indeed in its infancy, and while we briefly explore these issues in the Supplementary Materials (S1 File), principled guidelines for time window selection remains an exercise for further research. An exploratory test found that, for this particular dataset, including the beginning of the response (i.e., starting from 0 seconds after stimulus onset), yielded chance-level decoding performance. However, the retrospective analysis provided in the supplementary materials (S1 File) also revealed that decoding was generally best for time windows starting between 4-7s after the start of the stimulus block.

## Results

Decoding accuracy is averaged across all test infants to produce an overall decoding accuracy score: Infant-level decoding accuracy is expressed as the percentage of infants where the averaged multichannel patterns for the two conditions are correctly labeled (greater correlation for correct condition labels than the incorrect condition labels, see Eqs 4.1 and 4.2). Trial-level decoding accuracy is expressed as the average percent of correctly labeled trials across infants, where correct refers to cases where the correlation is higher for the correct condition label than the incorrect condition label (see Eq 5). We apply these tests to two datasets and ask whether these decoding methods reliably predict, on an infant-level or trial-level, the obtained infant fNIRS data. The output of the decoding scripts and our analysis code can be downloaded at https://github.com/laurenemberson/EmbersonZinszerMCPA_analysesFromPaper.

### Infant-level decoding

In Dataset #1, the infant-level multichannel patterns (averaged for each condition, Fig 2) were correctly labeled for 17 out of 19 infants (accuracy = 89%; see top panel of Fig 3, “Infant-level” data). In Dataset #2, the infant-level multichannel patterns for the two types of audiovisual trials were correctly labeled for 13 out of 18 infants (accuracy = 72%; see Fig 3, bottom panel). Because the cross validation procedure introduces dependencies between each infant that is tested against the group model, a parametric binomial test is not appropriate for testing the significance of these results. Instead, we performed a permutation-based test to create an empirical null distribution and determine the *p*-value for the observed decoding results. This procedure is described and illustrated in detail in the supplementary materials [32]. Decoding accuracy was statistically significant in both Dataset #1 (*p* = 0.001) and Dataset #2 (*p* = 0.048).

Thus, we find that the group model can successfully decode two stimulus conditions in an infant’s fNIRS data with high accuracy, determining whether a test infant is either seeing or hearing a stimulus (Dataset #1), and in the much more difficult contrast between different combinations of audio-visual stimulation (Dataset #2) where successful decoding cannot rely on stimulus modality exclusively. In Dataset #2, accurate labeling requires the correct discrimination of one audiovisual combination from another, such that only subtle patterns of cortical activation in the same or overlapping regions will distinguish these two conditions rather than broad spatial differences among channels (e.g., anterior-vs.-posterior or right-vs.-left), which we test in the univariate analyses presented later in this section.

### Trial-level decoding

For Dataset #1, labeling each trial independently for each test infant yielded 68% accuracy for Visual trials and 66% for Auditory trials (Fig 3, top panel). Each of these accuracy scores significantly exceeded chance (50%, see S1 File for the details of generating the null-hypothesis distribution and *p*-values; Visual: *p*<0.001; Auditory: *p*<0.001).

For Dataset #2, labeling each trial independently yielded 60% accuracy for Audiovisual-1 trials and 57% for Audiovisual-2 trials (Fig 3, bottom panel). While numerically above 50%, these accuracy scores did not robustly exceed chance (50%, Audiovisual-1: *p* = 0.054; Audiovisual-2: *p* = 0.125).

Thus, we find that the group model can successfully decode single trials for an infant who did not contribute to the group model (i.e., test infant). In the case of Dataset #1, both conditions were decoded significantly beyond chance. While for Dataset #2, trial-level decoding was around 60%, but did not reach the *p*<0.05 threshold for statistical significance.

### MVPA with subsets of channels: Investigating spatial-specificity

Since the current MVPA method works on multichannel patterns (i.e., vectors) of arbitrary (but consistent) length, it is also possible to perform the infant-level and trial-level analyses on subsets of the fNIRS channels, rather than using all available fNIRS channels as we reported in the previous sub-sections (10 channels).

Canonical MVPA analyses of fMRI data use voxel-stability and “searchlight” techniques to determine which voxels contain sufficient information to decode previously unlabeled trials [6,33]. Due to the small number of fNIRS channels and the large spatial extent of cortex over which each channel samples (~2 cm of infant cortex, [11]), fNIRS recordings from the two datasets are not conductive to a spatial searchlight analysis. However, the small number of channels permits an exhaustive combinatorial analysis of decoding accuracy for all possible channel subsets.

This subset analysis allows us to determine which channels make the greatest overall contribution to MVPA decoding accuracy. Specifically, we consider two interrelated issues:

1. How many channels should be included in a subset for maximum decoding accuracy? Does the inclusion of more channels result in better decoding?
2. Are some channels more informative than others? With smaller subset sizes, we test the varying contribution of each channel to decoding accuracy. These issues together allow us to tackle the question of whether this multivariate method can detect **spatial-specificity** in decoding accuracy. Foreshadowing our results, we will provide evidence that average decoding accuracy decreases with smaller subsets (e.g., reducing from 10 to 2 channels employed per subset). However, we can detect substantial variation of decoding accuracy across subsets and varying levels of decoding accuracy across channels indicating that some channels contribute more information to the decoding process than others. Together, these results provide evidence that spatially-specific decoding results can be obtained through MVPA.

In this subset analysis, we varied the number of channels included in the decoding analysis (*subset size*) from two channels to ten channels (i.e., the complete array, as reported in the foregoing results). For every subset size, _10_C_n_ possible combinations of *n* channels exist. For example, for a subset size of 2 channels, subsets would be channels 1 & 2, 1 & 3, 1 & 4, etc. We tested all subsets for infant-level decoding.

### Are there differences in decoding accuracy with subset size?

**Fig 4:**
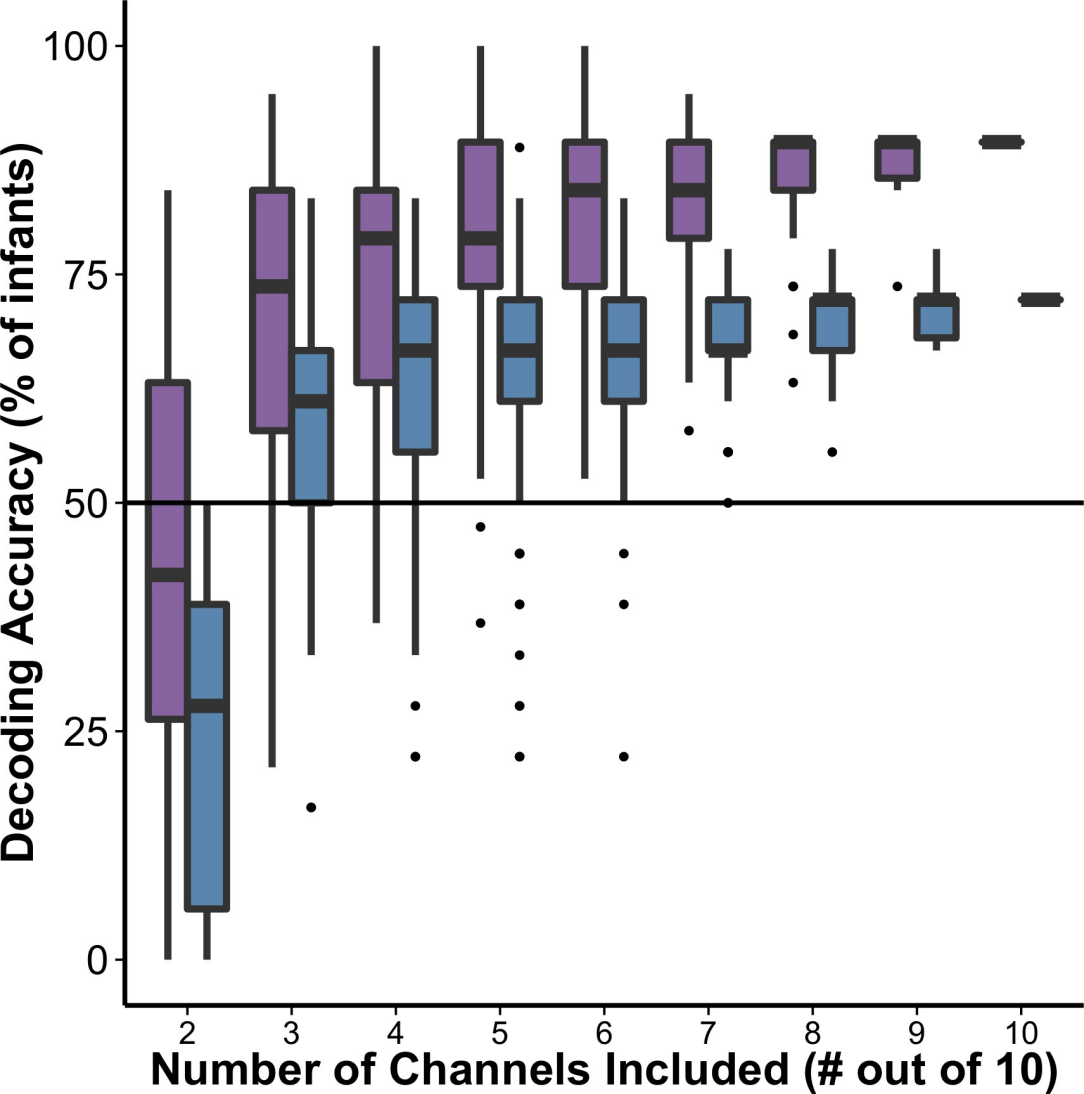
Decoding accuracy of infant-level activation patterns by subset size for Datasets #1 (purple boxes) and #2 (blue boxes). Far right, decoding using three most informative channels (most informative channels determined using subset size 2, Fig 3). Note: For the subset size of 10 channels, there is only one subset and so there is no range to estimate.

To determine how decoding accuracy changes with subset size, accuracy over all subsets was determined for each subset size and for both datasets. As shown in Fig 4, the difference in average decoding accuracy increases with larger subset sizes and peaks for both datasets when the subset size equals the total number of channels (subset size = 10). Thus, even though all channels are included across subsets, when subset sizes are less than the total number of channels (< 10), the maximum average decoding accuracy (across all subsets of the same size) improves as the number of channels simultaneously considered during decoding increases. Of course, the rise in decoding accuracy with numbers of channels included in the analysis is unlikely to monotonically increase *ad infinitum*. However, this point is not the one being made here as this method is intended to be applied to the fNIRS community and the developmental community in particular both of which are largely restricted to a small number of channels. Future research will be needed to consider the broader point of how decoding accuracy changes with numbers of channels beyond 10.

We estimated a logistic regression model to evaluate the following patterns of decoding accuracy for statistical significance:

1. differences in decoding accuracy between Datasets #1 and #2;
2. differences in decoding accuracy across channels;
3. the relationship between subset size and decoding accuracy.

In a mixed-effects logistic regression model [34], we examined infant-level decoding accuracy for each infant, for each subset, and for each channel across subset sizes 2 through 10. In other words, our model attempted to predict decoding accuracy before any of the data had been averaged. Thus, decoding accuracy is either correct, 1, or incorrect, 0, (based on whether the correlation between the infant-level multichannel pattern for the test infant and the group model is higher for the correct labels compared to the incorrect or switched labels, described in detail in Methods). Infants are treated as a random factor in the model to control for any variability across infants. See S1 File for more details.

First, we confirmed that the difference in accuracy across datasets was significant (coefficient = -1.61, *Z* = -36.28, *p* < 0.001). Second, by comparing across nested models using chi-squared tests, we confirmed that including channels in our model accounts for significant variance in decoding accuracy (χ^2^(333) = 7583.6, *p* < 0.001). This finding indicates that there are systematic decoding differences across channels. We also find that the additional inclusion of subset size in the model significantly increases its fit, indicating that there are differences in decoding accuracy across set size (χ^2^(370) = 12958, *p* < 0.001). Third, subset size (from 2 to 10) has a significant positive coefficient in this latter model (coefficient, 39.66, *Z* = 5.99, *p* < 0.001), confirming that with increases in subset size, there is an increase in accuracy. These results confirm that there are significant differences in decoding accuracy between datasets, across channels, and with increasing subset size.

### Are some channels more informative than others?

To test whether some channels are more informative than others, subset sizes of 2 to 10 channels were compared for infant-level decoding accuracy, with all possible combinations of channels tested in _10_C_n_ subsets. If all channels were contributing equal information to the group model, one would expect to see no or little variation across different subsets of channels. Instead, we see a large amount of variation across channels with an impressive range from greater decoding accuracy with all 10 channels to well below chance with fewer channels. Moreover, subset was demonstrated in our statistical model to explain a significant amount of decoding accuracy. In other words, the large amount variation across subsets that emerges as the size of the subset decreases provides some evidence that some channels are more informative than others.

**Fig 5:**
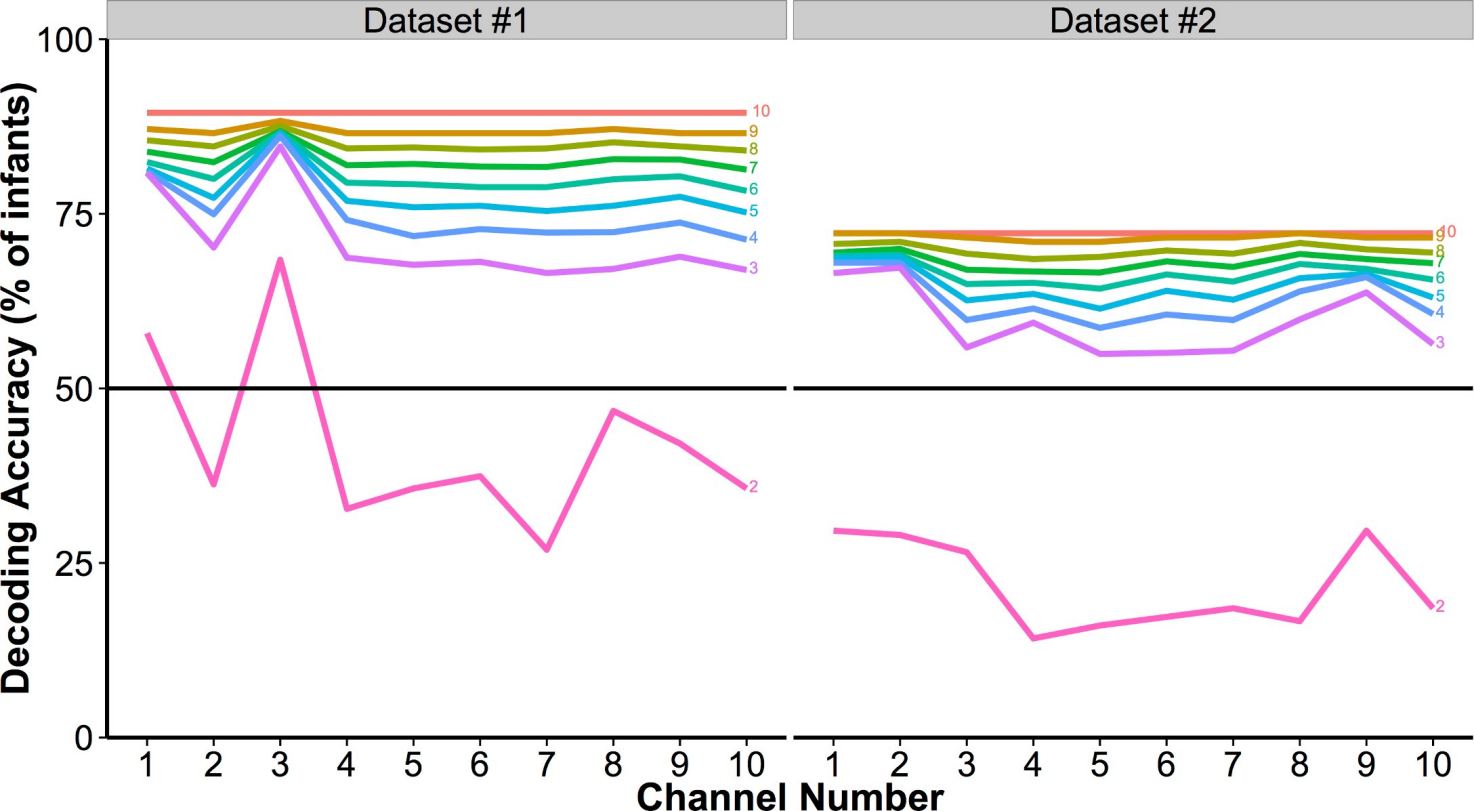
Accuracy for each of the 10 NIRS channels for Dataset #1 (left) and Dataset #2 (right) in different subset sizes (from 2 to 10 channels with each line labeled at the right with the subset size).

**Fig 6:**
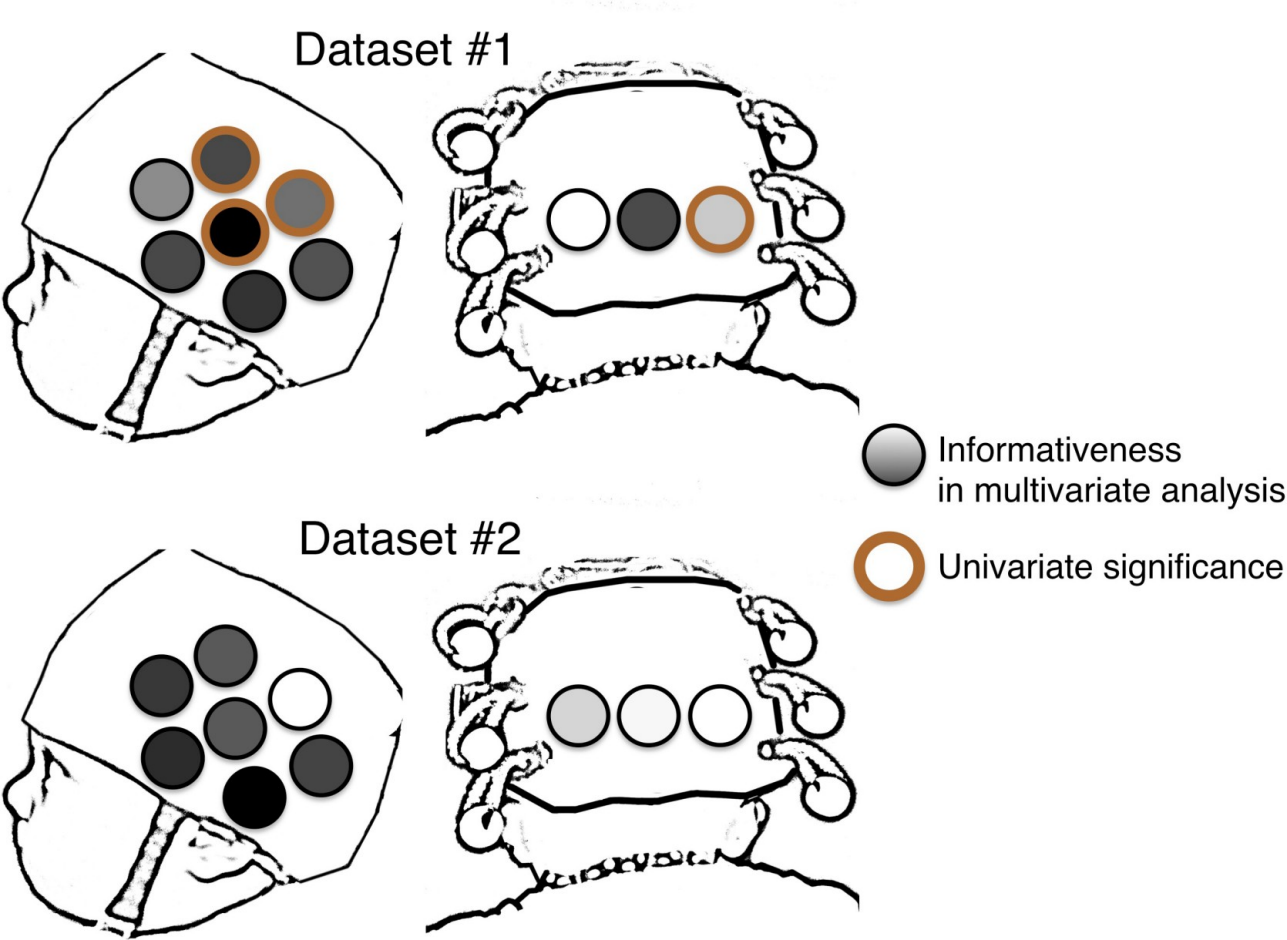
Comparison of the relative informativeness across channels from multivariate analysis (from dark to light, least to most informative respectively) and channels which exhibit a significant difference between the same two conditions in a univariate analysis. Across both datasets, only a single channel that exhibits a significant univariate response is one of the most informative channels in the multivariate analyses. In Dataset #2, not a single channel was significant for our univariate analysis but we achieve significant infant-level decoding in the multivariate analysis.

In addition, for each subset, the average decoding accuracy was assigned to each contributing channel. Once all subsets are decoded, each individual channel’s accuracy is defined as the average accuracy for all the subsets it participated in. Fig 5 presents average infant-level decoding accuracy for each of the 10 channels across subset sizes from 2 to 10 for both datasets. Subtle differences between channels become visually apparent as subset size decreases from 10 to 5 and more pronounced with subset sizes of 2 and 3. Fig 6 presents a depiction of the relative informativeness for each channel.

Interestingly, we find that although both Datasets involve stimulus conditions that contain auditory and visual information, there is some difference in which channels appear to be the most informative: for Dataset #1, the three most informative channels (numerically) are 1, 3 and 8; for Dataset #2, the three most informative channels are 1, 2, and 9. Information about the spatial location of these channels, their mean responses in each condition, and the correlations of these response in each channel can be found in the Supplementary Materials (S1 File) but generally, channels 1 through 3 are located in the occipital lobe spanning from V1 to LOC, with the remaining channels are distributed through the temporal cortex (5 channels) and the prefrontal cortex (2 channels). Thus, we find that as subset size decreases, there is more diversity in mean decoding accuracy across channels, with prominent differences revealed between channels in subset sizes 2 and 3. It is also interesting to note that for Dataset #1 while the differences between channels becomes more prominent as subset size decreases, the overall pattern of which channels are more informative compared to others remains stable. This is not the case for Dataset #2: Between subset sizes of 2 and 3, there are differences in which channels are (relatively) more or less informative, particularly in which channels are the least informative.

In sum, we present two pieces of evidence that spatial-specificity can be achieved in using this MVPA technique with fNIRS. First, we find relative informativeness of different channels across all subsets which provides evidence that not all channels are contributing the same information. Second, we find a large amount of variation in decoding accuracy across subsets of small numbers of channels indicating that not all subsets are equally able to decode across infants. Indeed, we see that some subsets have decoding accuracy that is (numerically) greater than decoding with all channels (subset size = 10). Again, this provides evidence that not all channels are equally informative and thus some spatial inferences can be made (i.e., which regions of the infant brain are contributing to decoding accuracy). See Supplementary Materials (S1 File) for some exploratory analyses selecting and validating small number of channels.

### Comparison to univariate analyses: Multivariate analysis distinguishes between conditions when univariate cannot

Multivariate analyses have the ability to exploit the relations between signals from different channels, whereas univariate analyses can only assess each channel individually. Thus, the greatest opportunity for multivariate analyses to reveal information beyond that obtainable by univariate approaches is when the task conditions stimulate broadly distributed regions of the brain at once. In the present study, Dataset #2 contains just such a pair of conditions (faces-and-music, versus fireworks-and-speech). Both conditions are audio-visual, but the specific nature of the audiovisual stimuli differs.

In a univariate analysis of Dataset #2, we compared average activation from 6 to 12.5 seconds after stimulus onset. No single channel exhibited significant differences between conditions after Bonferoni correction (0.05/10 or the number of channels). Thus, there are no robust univariate differences between these two audiovisual conditions. In contrast, our multivariate analysis was able to decode this dataset with high accuracy at the infant-level (> 75%, as depicted in Fig 3). There were three channels that were significant but did not survive correction (*p* < 0.05, Channels 8, 9, and 10). If we evaluate these channels for their relative informativeness in decoding, only Channel 9 appears to stand out and other highly-informative channels (1–3) are not identified as univariately significant. This comparison is illustrated in the lower panel of Fig 6: three channels were jointly able to provide highly accurate multivariate decoding (the three blue-only circles in the Dataset 2 section at the bottom of Fig 6), but there were no channels at all which provided significant univariate decoding (as shown by the absence of any brown rings in that panel). Thus, in trying to distinguish between two types of stimuli, which were both audio-visual but whose content differed (faces-and-music, versus fireworks-and-speech), multivariate analysis was able to distinguish between conditions when univariate could not.

The literature on fMRI decoding has numerous direct comparisons of univariate statistics and multivariate decoding methods (e.g., [19]). At least one previous study of fNIRS decoding has demonstrated the effectiveness of decoding in the absence of significant univariate results [27], but that study measured nine-year-old children, a subject group far more cooperative than the infants of our present study, and hence allowed for many more stimulus-presentation trials and much less noisy data than we are using here. Thus, while the benefits of employing MVPA for fNIRS go beyond the benefits of statistical power (see the Introduction), it is important to consider whether MVPA has the potential to uncover neural differences beyond univariate statistics.

We also compared univariate and multivariate results in Dataset #1. We chose a standard univariate approach (single-channel mean-based comparisons between conditions with statistical corrections) to compare to our current methods. This choice does not maximize the statistical similarity between the methods (which would require non-standard statistical methods for univariate tests) but does maximize comparison between the current approach and those already employed in the literature. Average activation was compared between 4 and 9 seconds after stimulus presentation [15]. As we expected, there are channels that survive correction in the comparison of unimodal audio and visual stimuli. Specifically, 4 channels exhibited significant differential activity (Channels 1, 7, 9, 10, *ts*(18) > |3.34|, *ps* < 0.0036). Interestingly, these are not the same channels that were the most informative in decoding (i.e., Channels 1, 3 and 8; please see the fully cross-validated test in S1 File). The channel with the highest relative decoding accuracy (Channel 3) did not exhibit significant differentiation of the two conditions even before statistical correction. An additional 3 channels exhibited significant differences (*p* < 0.05) but did not survive correction (Channels 4, 5 and 8). Thus, even in the case where infants were presented with unimodal audio and visual stimuli, univariate statistics and MVPA decoding yield different (but highly significant) results, suggesting that these analytic approaches provide different types of information.

## Discussion

We present evidence of significant decoding of the infant brain using a novel multivariate analysis of fNIRS data from two infant datasets. These findings are notable for demonstrating a simple, effective multivariate method for fNIRS data with highly accurate decoding of neural responses across infants that either complements or surpasses univariate analyses. Specifically we proposed a correlation-based analytic framework for conducting a multivariate analysis on a small set (n = 10) of NIRS channels. The resultant multivariate pattern analysis (MVPA) reliably decoded which of two stimulus conditions was present (i.e., the average pattern of response across channels) both at the infant-level and at the trial-level (i.e., the average pattern for an infant across all trials and the average pattern for a single trial, respectively). Infant-level performance was robust across two datasets, which included both unimodal and multimodal stimuli. Trial-level performance was reliable for unimodal stimuli, and despite not achieving statistical significance, trial-level prediction accuracy in the multimodal stimuli (around 60%) was strongly suggestive of subtle differences in the sensory properties of the two stimulus conditions. Future trial-level discrimination may simply require more statistical power (i.e., larger sample size of infants). Nevertheless, decoding accuracy using MVPA was highly robust under for decoding across infants in both unimodal and multimodal stimulus conditions. This result is especially impressive given the many hurdles that must be overcome when gathering fNIRS data from infants (e.g., limited number of channels, large spatial extent of sampled cortex per channel, small number of trials contributed per infant, greater intersubject variability).

The subset analyses suggest that MVPA methods benefit from including a greater numbers of channels, highlighting the multivariate aspect of this method. Further, this analysis revealed **differential informativeness of individual channels** to MVPA decoding accuracy. Specifically, we find that if the number of channels contributing to a multivariate analysis decreases, there is an increase in the variance across subsets, indicating that some combinations of channels achieve nearly identical decoding performance to the full set of channels and other combinations of channels have very poor decoding accuracy. Moreover, the average decoding accuracy for a given channel varies across channels. These two findings provide evidence that not all channels are contributing equal information to the decoding and provides some spatially-specific information about representations in the infant brain can be recorded using fNIRS and decoded using MVPA. Moreover, both of these findings validate the multivariate method as a tool for identifying pattern-based neural correlates of cognition: The decrease in average decoding accuracy with smaller subset sizes establishes that the current analysis method utilizes patterns of activation distributed across channels and thus suffers when fewer of these channels are contributing to the observed pattern of activation. It also follows that not all channels contribute equally to decoding, and thus channel-wise analyses indicate differences in specialization or engagement of specific cortical regions to the current task, which is detectable using MVPA.

The more informative channels identified in the present study were widely distributed across the cortical surface including occipital, temporal and frontal regions. Moreover, the relative pattern of informativeness across channels was not the same across datasets. These findings highlight the value of MVPA for pooling information across the brain without spatial constraint. The evaluation of subset informativeness opens a window on exploring the continuum of modular (i.e., cluster-based) versus distributed neural architectures, including how such architectures might be biased before birth and/or emerge with exposure to postnatal experience. This inference about spatial localization intersects with the goals of univariate analyses, which focus on clusters of channels (i.e., an ROI approach). However, a direct comparison of univariate and multivariate methods in this study revealed that MVPA elucidates aspects of the hemodynamic signal that are missed in standard univariate tests. In the multimodal dataset (Dataset #2), significant infant-level decoding is successful using a subset of informative channels while univariate methods fail to find any significant differences on these (or indeed, any) channels. Moreover, in both datasets, the more informative channels did not strongly overlap with the channels that exhibited significant univariate results. In fact, only a single channel both survived statistical correction in univariate tests and was one of the more informative channels for decoding performance (Channel 3 in Dataset #1). Thus, on two fronts we find that MVPA notably extends results found using univariate methods: In the absence of significant univariate results, decoding is still possible (Dataset #2) and individual channels can be identified as supporting decoding while yielding no significant univariate results and vice versa. However, it is important to note that for both univariate and multivariate methods, it is possible that differential response/informativeness could arise from signals that are correlated with the contrasts of interest. This issue must be dealt with in the experimental design and cannot be readily dealt with in the analyses regardless of whether they are multi- or univariate.

It is notable that decoding was conducted between-infants (i.e., based on a group model that did not include data from the test infant). It is an important area of future empirical work to investigate the nature of the neural patterns or representations supporting this between-subject decoding. Intuitively, between-subjects decoding is likely to be relying on more coarse-grained patterns than within-subjects decoding because of the variability of representations, neural specialization and, pragmatically, the localization of NIRS channels across subjects. However, previous work with adult fMRI decoding has demonstrated that adults do share fine-grained neural representations when they are considered in similarity space [35].

Given this initial proof of concept that multivariate methods can be used successfully to decode two stimulus conditions from the fNIRS data of 6-month-old infants and provide findings beyond univariate tests, there are a large number of questions that can be tackled which were hitherto inaccessible to fNIRS studies using univariate methods. For example, rather than asking whether cortical region X is more activated by a particular type of stimulus (e.g., faces) than some other comparison stimulus (e.g., houses), one can ask two more subtle questions. First, is there a *pattern* of activation that, even in the absence of a difference in mean activation for the two stimulus conditions, indicates reliable decoding of that stimulus difference? Second, given a reliable pattern of decoding, what is the spatial distribution of fNIRS channels that are *informative* for that decoding process? Answers to these two questions, which our findings now bring into the realm of possibility, will enable researchers who study the *development* of the human brain to begin to propose and evaluate more sophisticated models of the neural mechanisms of cognition. In addition, by relating the patterns of fNIRS activations among stimuli that vary along known dimensions, one can expand MVPA to ask how higher-level stimulus dimensions are decoded by the brain. Now that MVPA has been confirmed as a viable method in infants, future use of Representational Similarity Analysis promises to be a fruitful avenue for investigation, such as implementing methods for more than two conditions, as described by [21] and [35].

Aslin et al.'s [12] review of fNIRS contribution to developmental research highlighted the imminent need to extend multivariate methods developed in fMRI to fNIRS. Although some previous studies have implemented machine learning techniques to classify fNIRS responses, these multivariate approaches have not yet been widely adopted by the fNIRS community, and we have argued that there are significant barriers to doing so both computationally and interpretatively. In this paper and the supplementary material, we offer a simple, transparent method for decoding fNIRS data in an early developmental population. The MATLAB code for implementing these analyses is publicly available, flexible enough to easily accommodate various experimental configurations (e.g., different numbers of fNIRS channels or subsets of channels), and can automatically extract analysis-relevant details from HomER data files [28]. The availability and transparency of the methods and code will also allow researchers to make changes that expand the use of multivariate analyses in their fNIRS research.

## Supporting information

S1 File Supplementary material, Results, Tables and Figures. (DOCX)
