# Supplementary material for: Decoding the infant mind: Multivariate pattern analysis (MVPA) using fNIRS
Source: PLoS One. 2017 Apr 20;12(4):e0172500. doi: 10.1371/journal.pone.0172500 (PMC5398514; doi:10.1371/journal.pone.0172500)
Supplement: S1 File — (DOCX) [file pone.0172500.s001.docx]

**Supplementary Materials**

**NIRS Channel Locations: MR-coregistration**

Across both datasets, the same NIRS cap configuration was employed. MR co-registration for this cap configuration has been conducted for over 100 infants to reveal the average cortical localization for each NIRS channel. Table 1, adopted from Emberson et al., (2015), presents the localization for a representative subset of these infants and including infants in Dataset #1. See Emberson et al., (2015) for detailed descriptions of the MR-coregistration methods employed.

It is important to note that while the MR-coregistration methods employed provide evidence of the average spatial location, there are certainly differences across individual infants in the precise cortical regions being recorded from a given channel for a number of reasons. First, the NIRS cap is a fixed size and configuration so, despite efforts to standardize placement of the cap for each infant, any change in head size will result in differences in the cortical regions being recorded for each channel. Second, individual differences in neuroanatomy are not accessible to fNIRS because unlike fMRI no anatomical image of each brain is able to be collected. Given that our application of MVPA to fNIRS employs between-subjects decoding, it is a strong assumption of the method that the same channel is recording from the same cortical regions across the dataset. However, given the large size of each fNIRS channel (~2cm of cortex), it is not clear how much these variations matter for MVPA decoding or, indeed, any fNIRS data analysis. Of course, better spatial specificity in the recordings will allow MVPA to reveal more precise spatial specificity in the dataset, and if a researcher makes no attempt to control or at least quantify the locations of the fNIRS channels, this will certainly reduce the accuracy of decoding. However, we believe that the major constraint on spatial specificity for MVPA is the size of the fNIRS channels and the lack of collection of individual neuroanatomical images for each subject.

**Permutation-Based Significance Tests**

The assumptions for the binomial test are not satisfied in *n*-fold cross validation (i.e., leave-one-infant-out cross validation for all *n* infants) due to the non-independence of each fold (violates the *i.i.d.* assumption of a parametric test). Thus it is necessary for us to generate an empirical null distribution composed of either all the possible outcomes under the null hypothesis or a large, random sample of those outcomes. This null distribution is estimated by permuting the labels on each subject’s activation patterns for the two conditions and computing the distribution of decoding accuracies across many (e.g., 10,000) such permutations (see Nichols & Holmes, 2003 for background).

*Infant-Level Decoding*

Because there are two conditions, the number of possible label permutations in this dataset is 2^*n* where *n* is the number of subjects. Because the infant-level decoding is computationally fast (<1 sec to complete the entire *n-*fold cross validation), we were able to explore all possible permutations for each dataset (2^19 for Dataset #1 and 2^18 for Dataset #2). In this case, the null distributions represent *all* possible label permutations, and the null hypothesis is that infant-level decoding accuracy with the correct condition labels does not significantly differ from randomly assigned labels.

Figure S1 depicts frequencies (histogram divided by total number of permutations) for the accuracy of the infant-level permutation tests of Dataset #1 (left panel) and Dataset #2 (right panel). *P*-values for the observed decoding accuracies (Dataset #1: 17/19 infants correctly labeled; Dataset #2: 14/18 infants correctly labeled) are computed by measuring what proportion of observations are equal to or greater than the observed decoding accuracy on the null distribution. For reference, all values on the distribution with *p*<0.05 are highlighted in red.


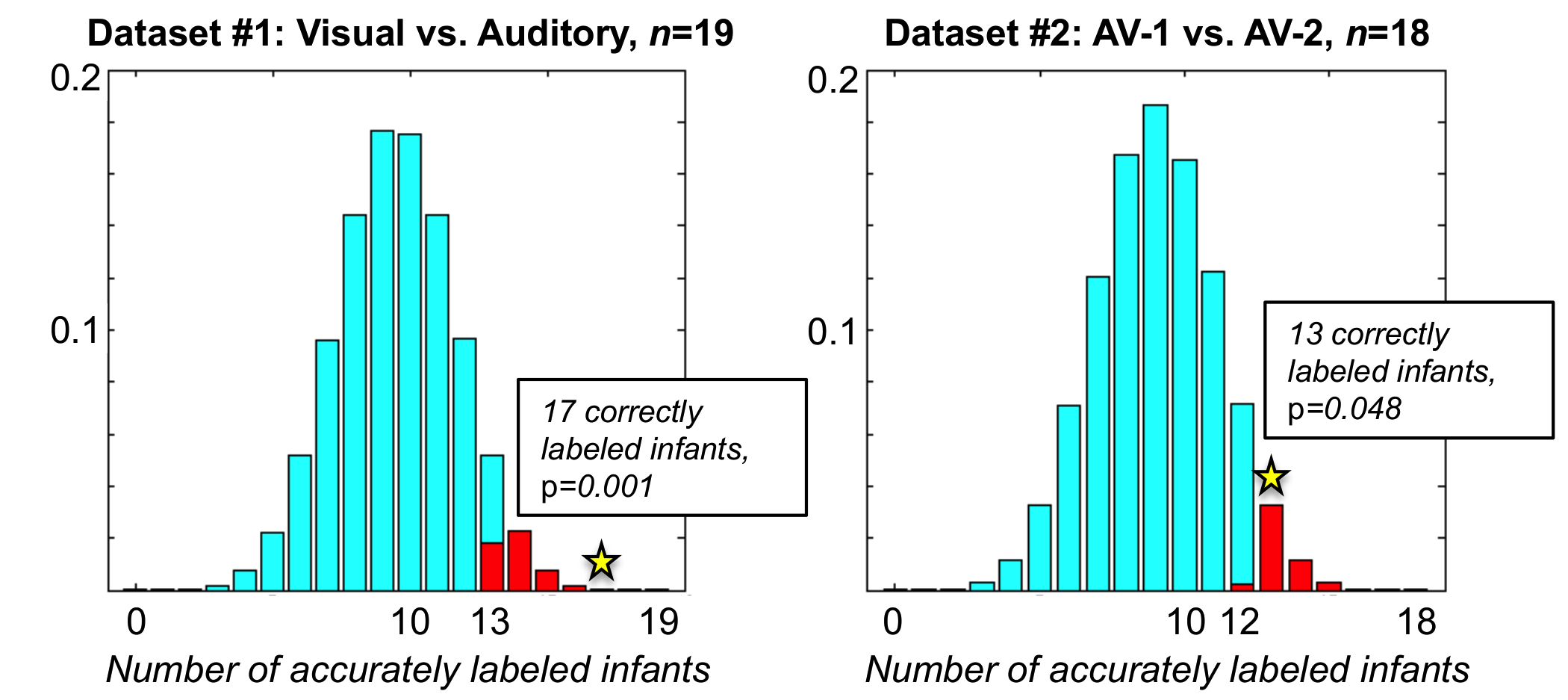


Figure S1: Null distributions for infant-level decoding in Dataset #1 and Dataset #2. Values with empirical *p*-value less than 0.05 are highlighted in red, and observed accuracies reported in Results section are indicated with a star and labeled.

*Trial-Level Decoding*

The permutation procedure for trial-level decoding was the same as infant-level decoding, but instead of measuring the number of correctly labeled infants, the number of correctly labeled trials for each condition was averaged across infants in the cross validation (following the same method as computing actual accuracy described in the Methods). Because trial-level decoding requires greater computation time (approximately 2 seconds per *n* folds), searching the entire set of possible permutations was computationally infeasible. Instead, we randomly sampled 10,000 permutations from the set of all possible permutations. Mean trial-level accuracy across infants is calculated for each condition in each permutation. The null distribution thus represents the average trial-level decoding accuracy when labels are randomly assigned.

Figure S2 depicts the null distributions for each of the two conditions in Datasets #1 (top two panels) and #2 (bottom two panels). *P*-values for the observed decoding accuracies (Dataset #1: Condition 1 68%, Condition 2 66%; Dataset #2: Condition 1 60%, Condition 2 57%) are again computed by measuring what proportion of observations are equal to or greater than the observed decoding accuracy on the null distribution. For reference, all values on the distribution with p<0.05 are highlighted in red.


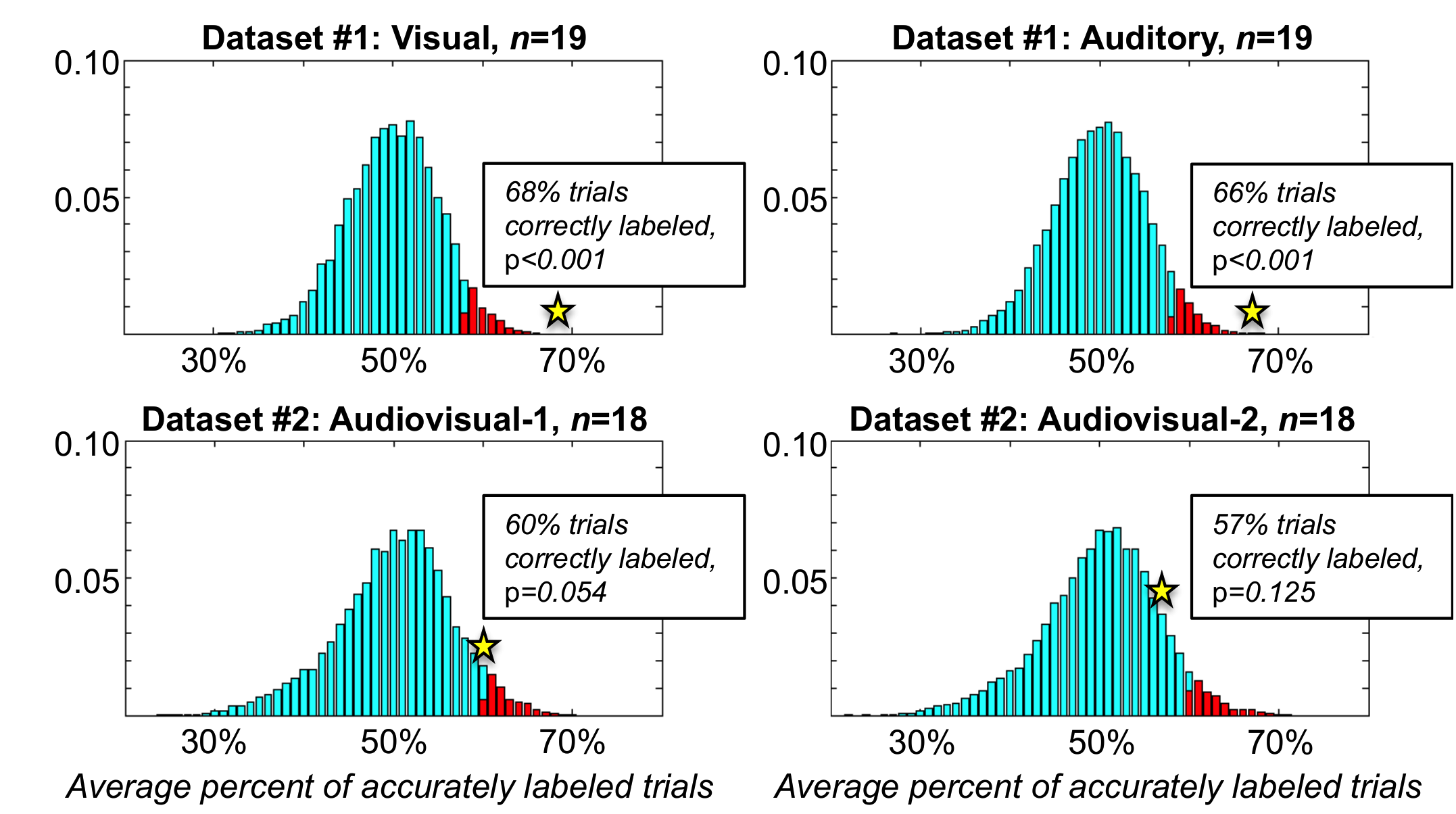


Figure S2: Null distributions for trial-level decoding for each condition in Dataset #1 and Dataset #2. Values with *p*<0.05 are highlighted in red, and observed accuracies reported in Results section are indicated with a star and labeled.

**Channel selection and cross-channel correlations**

The subset analyses presented in the main text of this paper showed that reducing the number of channels represented in each multivariate activation pattern decreased the average decoding accuracy (see Fig. 4). However, we also found that not all channels performed equally poorly in small subsets (see Fig. 5), suggesting that the informational content encoded in these channels varied in specific ways relevant to the stimulus conditions. MVPA provides tools for identifying what informational content in the data contributes towards successful decoding, and here we ask (1) which channels make the greatest contributions towards accurate decoding and (2) whether these channels tended to respond similarly (containing information about the same signal) or differently (containing information about different signals, different aspects of the stimulus) relative to one another.

*Identifying the best channels for decoding*

One way to estimate the relative informativeness of channels is by observing their average decoding performance across smaller subset sizes. If the channels that show the highest average performance actually carry the most useful information for decoding, we would expect these channels to perform well in combination. In other words, is the decoding accuracy for a set of the three most informative channels higher than what one would expect based on a random sampling of any three channels? If including only the three best channels boosts decoding accuracy, this would be evidence that these specific channels capture specific inter-regional differences in the stimulus-relevant information encoded in the infant brain.

For this analysis, we operationally define the most informative channels as the channels with the highest average decoding accuracy at the subset size of 2 (see Fig. 5 for example: Dataset #1: channels 1, 3, & 8; Dataset #2: channels 1, 2, & 9). In order to test the performance of these channels, it is necessary to include an additional layer of cross-validation to avoid re-sampling the same data in both the channel selection stage and the top-channels decoding stage (the “double-dipping” problem). To achieve this nested cross-validation, we first excluded one infant from the dataset and performed the cross-validated subset analysis (as described in Fig. 4) on *n*-1 infants. Thus each fold of the subset analysis applies *n*-2 infants in the group model toward decoding one infant, repeated *n*-1 times for the entire set of infants (besides the initial left-out infant). Based on this subset analysis, we selected the best three channels to combine in a single subset (size=3) and decode the initially left-out infant. This process is iterated over all *n* infants, yielding the second layer of *n-*fold cross-validation.

This nested cross-validation procedure for channel selection in Dataset #1 reproduced the pattern described in the main text: Channels 1, 3, and 8 were selected as the top performing channels in every fold of the cross validation. A subset combining these three channels yielded accurate decoding in 17/19 infants. Thus, these three channels appear to carry about as much information relevant to discriminating between the two stimulus conditions as the entire set of 10 channels, with reliable localization across infants. The content of that information is briefly examined in the next section.

The same procedure for Dataset #2 produced more complicated results. The channel search procedure identified channels 1 and 9 as top channels in all 18 folds, and channel 2 as one of the top three channels in 14 out of 18 folds (channels 3, 8, or 10 out-performed channel 2 in the remaining four folds). However, in the 14 folds that included the majority-consensus top three (1, 2, and 9), only 8/14 left-out infants were accurately decoded, approximately chance performance. The alternative sets composed of channels 1 and 9 with either 3, 8, or 10 were also at chance: 2/4 folds. These results seem to suggest that on one hand, channels 1, 2, and 9 reliably encode information relevant to discriminating between the two integrated audiovisual stimuli, but that on their own, they are inadequate for reliable discrimination between the two conditions, and a larger subset may be necessary for accurate decoding.

*Informational content of individual channels*

Figure S3 shows the cross-channel correlations for each participant’s multivariate response patterns. These responses were averaged across trials in the same condition for each subject and correlated between subjects. In other words, across subjects, how do the channels’ mean responses tend to compare with other channels?

The left and center panels of Figure S3 depict the cross-channel correlations for all channels. The far right panels of the figure depict the average response amplitude in the three top performing channels for each Dataset (subset decoding accuracy from the previous section notwithstanding). In both Datasets #1 and #2, the cross-channel correlations reflect the anatomical locations of the channels over the cortex. The posterior channels 1, 2, and 3 (roughly, occipital lobe) were strongly correlated with each other in Dataset #1 (top row) and far less correlated with the lateral channels 4-10 (recording from the frontal, temporal and parietal lobes). Likewise, the lateral channels correlated with one another more strongly than with the posterior channels. This pattern was less clear for the multimodal stimuli in Dataset #2, but appears to hold overall.

The mean responses from the subsets of three channels in each Dataset support this pattern, and it further suggests that the occipitial/frontal-temporal-parietal dissociation was important for successfully decoding both the modality-specific stimuli (Visual vs Auditory) and the integrated stimuli (AV-1 vs. AV-2). Dataset #1 (top right panel) indicates that channels 1 and 3 responded more strongly to the Visual condition than the Auditory condition, while channel 8 responded more strongly to the Auditory condition than the Visual condition. In Dataset #2, channels 1 and 2 likewise responded similarly across conditions (higher in AV-1, lower in AV-2) while the lateral channel 9 responded in the opposite direction. Overall, it appears that an integration of data from both of these broad cortical regions is necessary to produce the best decoding results.

Figure S3: Cross-channel correlations for Datasets #1 and #2. Left and center panels depict the Pearson *r* correlation between channels’ mean responses in each participant, quantifying how similarly channels tend to behave to one another when observed in multiple participants. The far right panels plot the mean response amplitude used for decoding in three selected channels, visualizing the information that was leveraged to classify conditions in the leave-one-infant-out decoding tests.

**Models of Decoding Accuracy**

In subsection “Are there differences in decoding accuracy with subset size?”, the data modeled is the decoding accuracy (1/0) for each infant, for each subset, for each channel, for each dataset which allows us to determine whether there are differences in decoding accuracy across dataset, across channels, and what the relationship is between subset size and decoding accuracy. The employed the glm function in R. Here, we include pseudo-code to illustrate the details of the model. We've made both this data and the code available for more detailed analysis, replication, and extension. Results and coefficients of these models are reported in the main text of the paper.

*Subset Channel Infant Accuracy Subset Size Experiment*

1 1 1 0 2 Dataset #1

2 1 1 0 2 Dataset #1

3 1 1 0 2 Dataset #1

4 1 1 0 2 Dataset #1

5 1 1 0 2 Dataset #1

6 1 1 0 2 Dataset #1

We start by determining whether there are differences in decoding accuracy across datasets (with Dataset #1 set as the intercept).

model1 <- glm(accuracy ~ dataset*Ss^[[1]](#footnote-1)^,

decodingaccuracydata, family = ‘binomial’)

When then add the additional factor of channel to the model (channel #1 as the intercept) and evaluate whether this addition significantly predicts more of the variance as a significant increase in model fit would indicate that decoding accuracy is not equal across channels.

model2 <- glmer(accuracy ~ dataset * channel*Ss,

decodingaccuracydata, family = ‘binominal’)

anova(model1,model2,test=’Chisq’)

We then determine whether the addition of subset size (numeric predictor) significantly improves the model and if so what kind of relationship subset size has on decoding accuracy (e.g., is it a significant positive relationship as indicated graphically).

model3 <- glmer(accuracy ~ dataset*channel*subsetsize*Ss,

decodingaccuracydata, family = ‘binominal’)

anova(model2,model3,test=’Chisq’)

See https://github.com/laurenemberson/EmbersonZinszerMCPA_analysesFromPaper for code to implement these models as well as the decoding accuracies from the two datasets reported in this paper.

**Effects of time window selection**

In the main text of this paper, we outline a rationale for selecting the time windows we used for each dataset, based on a visual inspection of the responses. The resulting time windows are defined by two parameters: *start time* after the onset of the stimulus and *duration* (the size of the window). While the time windows we used for this study yielded very encouraging results, the best way to choose a window remains an open question.

To explore this issue, we repeated the MVPA analyses for infant-wise and trial-wise classification at onset times every 0.1s from 0 to 12s after stimulus onset, depicted on the horizontal axes of Figures S4-S7 (below). At each onset time, we tested a range of window durations, both shorter and longer than the windows presented in the main text of this manuscript. Decoding accuracies were smoothed across the start-times using a 1 second window to reduce random variation and aid interpretation of the results. (It is unlikely that accuracy differences between 0.2s and 0.3s represent actual time window effects.) The encircled point on the figures denotes the original time window reported in this manuscript.

*Dataset #1: Event-related time window analysis*

The selected window start time of 0 seconds produced sub-optimal infant-wise decoding, and accuracy gradually increases with later start times for several of the window durations (see Figure S4). The 0-1s interval after stimulus onset is noticeably worse for all window durations. This time period may contain random variation prior to a hemodynamic response to the stimulus or carry over from the previous trial/block. The 2.5s duration gives a finer-grained view of when the decodable response probably occurred. The selection of a 10s duration (window size) appears to have been a reasonably good choice across the range of possible start times.

Figure S4: Infant-level decoding accuracy in Dataset #1 for time windows starting between 0-12s after stimulus onset. Multiple window durations are depicted as differently colored lines. The window in this study is denoted as an encircled dot (start time: 0s, duration: 10s). Accuracy data have been smoothed over a 1s window.

Trial-level accuracy (Figure S5) was also sub-optimal at a start time of 0s, and steadily rose to peak at 0.5s (Condition 1) or at 2s (Condition 2). Around these early times, the 10s window performed on par with other window durations. Condition 2 decoding appeared to improve at two later start times, similar to the infant-level decoding.

Figure S5: Trial-level decoding accuracy in Dataset #1 for time windows starting between 0-12s after stimulus onset and multiple durations.

*Dataset #2: Block design time window analysis*

We found that the selected start time of 6.0s for Dataset #2 was associated with strong decoding performance relative to earlier start times. The 2.5s duration data provided very little information for decoding until later, suggesting that the information of interest was not localized exactly in this window, but closer to 5.5-7s after stimulus onset. A second and very sharp peak in decoding accuracy occurred around 10s after stimulus onset for all duration values. The 2.5s duration window alone peaked later at 10.7s, suggesting the interesting effect occurs between 11-13.5s after stimulus onset, illustrated in the Figure S6.

Figure S6: Infant-level decoding accuracy in Dataset #2 for time windows starting between 0-12s after stimulus onset. Multiple window durations are depicted as differently colored lines. The window in this study is denoted as an encircled dot (start time: 6.0s, duration: 6.5s). Accuracy data have been smoothed over a 1s window.

Trial-wise decoding reflected similar patterns in both audiovisual conditions. Two relative maxima occurred for each condition around 5-6s after stimulus onset and again around 1-2s after stimulus offset (that is, 9-10s after stimulus onset). For both conditions, the 6.5s window produced the best (AV-1) or comparable-to-best (AV-2) decoding accuracy. In this sense, our selection of the 6.5s time window starting at 6.0s after stimulus onset was surprisingly advantageous. As pictured in Figure S7 with the encircled points, the reported window is very close to the peak performance for any 6.5s duration window, and both AV-1 and AV-2 would have achieved statistical significance (*p*<0.05) at slightly earlier start times (around 5.5s after stimulus onset).

Figure S7: Trial-level decoding accuracy in Dataset #2 for time windows starting between 0-12s after stimulus onset and multiple durations.

*Discussion*

Through the time window searches, we explored the effects of the start time and duration parameters on decoding accuracy. We can examine these search results to find windows that produce the best decoding, but we have also restricted ourselves from returning to the original datasets to repeat the hypothesis tests at the seemingly optimal time windows. However, the patterns observed in this analysis may generalize to new decoding studies.

Based on Dataset #1, we propose that starting the time window around 1s after stimulus onset is likely to improve decoding accuracy in new, unrelated datasets. This advantage is consistent with the brief post-stimulus delay (or even dip) in standard models of a hemodynamic response function. The analysis for Dataset #2 also generally supports the delay start time, with further allowance for the block design. While the event-related design seemed to be optimized by a very brief delay, the block design was best decoded around 5-6s after stimulus onset. This observation is consistent with the purpose of using a block design: When the response from a single stimulus is expected to be relatively small, experimenters block several stimuli together to gradually increase the response amplitude.

Reproducing these decoding analyses with new data will be necessary to determine whether these effects apply more broadly across different types of stimuli and experimental designs.

1. In R, formulae written indicating the interaction of two factors are, by default, considered as both the two main effects first and then the interaction. I.e., accuracy ~ dataset*Ss is actually run as accuracy ~ dataset + Ss +dataset*Ss [↑](#footnote-ref-1)
